# Increase in prevalence of current mental disorders in the context of COVID-19: analysis of repeated nationwide cross-sectional surveys

**DOI:** 10.1017/S2045796020000888

**Published:** 2020-09-29

**Authors:** P. Winkler, T. Formanek, K. Mlada, A. Kagstrom, Z. Mohrova, P. Mohr, L. Csemy

**Affiliations:** 1 National Institute of Mental Health, Topolová 748, 250 67, Klecany, Czech Republic; 2 Health Service and Population Research Department, Institute of Psychiatry, Psychology and Neuroscience, King's College London, David Goldberg Centre, De Crespigny Park, London SE5 8AF, UK; 3 Faculty of Medicine in Pilsen, Charles University, Husova 3, 301 00 Pilsen, Czech Republic; 4 Third Faculty of Medicine, Charles University, Ruska 87, 100 00 Prague, Czech Republic

**Keywords:** Anxiety, depression, COVID-19, mental disorders, prevalence, SARS-CoV-2, suicide risk

## Abstract

**Aims:**

The United Nations warned of COVID-19-related mental health crisis; however, it is unknown whether there is an increase in the prevalence of mental disorders as existing studies lack a reliable baseline analysis or they did not use a diagnostic measure. We aimed to analyse trends in the prevalence of mental disorders prior to and during the COVID-19 pandemic.

**Methods:**

We analysed data from repeated cross-sectional surveys on a representative sample of non-institutionalised Czech adults (18+ years) from both November 2017 (*n* = 3306; 54% females) and May 2020 (*n* = 3021; 52% females). We used Mini International Neuropsychiatric Interview (MINI) as the main screening instrument. We calculated descriptive statistics and compared the prevalence of current mood and anxiety disorders, suicide risk and alcohol-related disorders at baseline and right after the first peak of COVID-19 when related lockdown was still in place in CZ. In addition, using logistic regression, we assessed the association between COVID-19-related worries and the presence of mental disorders.

**Results:**

The prevalence of those experiencing symptoms of at least one current mental disorder rose from a baseline of 20.02 (95% CI = 18.64; 21.39) in 2017 to 29.63 (95% CI = 27.9; 31.37) in 2020 during the COVID-19 pandemic. The prevalence of both major depressive disorder (3.96, 95% CI = 3.28; 4.62 *v.* 11.77, 95% CI = 10.56; 12.99); and suicide risk (3.88, 95% CI = 3.21; 4.52 *v.* 11.88, 95% CI = 10.64; 13.07) tripled and current anxiety disorders almost doubled (7.79, 95% CI = 6.87; 8.7 *v.* 12.84, 95% CI = 11.6; 14.05). The prevalence of alcohol use disorders in 2020 was approximately the same as in 2017 (10.84, 95% CI = 9.78; 11.89 *v.* 9.88, 95% CI = 8.74; 10.98); however, there was a significant increase in weekly binge drinking behaviours (4.07% *v.* 6.39%). Strong worries about both, health or economic consequences of COVID-19, were associated with an increased odds of having a mental disorder (1.63, 95% CI = 1.4; 1.89 and 1.42, 95% CI = 1.23; 1.63 respectively).

**Conclusions:**

This study provides evidence matching concerns that COVID-19-related mental health problems pose a major threat to populations, particularly considering the barriers in service provision posed during lockdown. This finding emphasises an urgent need to scale up mental health promotion and prevention globally.

## Introduction

As COVID-19 became a global pandemic, countries responded with nationwide lockdowns in attempt to slow and prevent further spread of the virus. With over half of the world population on some form of lockdown in April 2020, mental health of populations became a growing concern as individuals faced unprecedented levels of established mental health risk factors including social isolation, stress and anticipated economic hardship (Monroe and Simons, 1991; Mazure, 1998; Hammen, 2004; Ahnquist and Wamala, 2011; Matthews *et al*., 2016; Herbolsheimer *et al*., 2018; Economou *et al*., 2019; Brooks *et al*., 2020). These risk factors not only disproportionately affect individuals with a history of mental health problems (Hao *et al*., 2020; Yao *et al*., 2020), high-risk groups such as health care workers (Kang *et al*., 2020; Liu *et al*., 2020; Lu *et al*., 2020), COVID-19 patients and survivors (Zhang *et al*., 2020*a*), individuals with pre-existing chronic diseases (Ohliger *et al*., 2020; Wang *et al*., 2020*b*) or unemployed individuals (Zhang *et al*., 2020*b*), but also could trigger the onset of mental disorders in previously healthy populations. Alarming statements by public health experts and the United Nations have expressed the concern that COVID-19 could contribute towards a major global mental health crisis (Galea *et al*., 2020; UN, 2020).

Evidence on the prevalence of COVID-19-related mental health problems is emerging. A nationwide online survey of participants from China recruited through convenience sampling (*n* = 1210) reported that 16.5% of individuals exhibited severe depressive symptoms, and 28.8% moderate to severe anxiety symptoms (Wang *et al*., 2020*a*). Another nationwide online survey using convenient sampling in China estimated that the prevalence of anxiety disorders, depressive symptoms and reduced sleep quality was 35.1, 20.1 and 18.2%, respectively (Huang and Zhao, 2020). An online study (*n* = 4872) from Wuhan, China, found a 48.3 and 22.6% prevalence of depression and anxiety among the general adult population (Gao *et al*., 2020). The largest study conducted in China (*n* = 52 730) found 35% of respondents experienced psychological distress as assessed by the COVID-19 Peritraumatic Distress Index (Qiu *et al*., 2020). Nationwide studies from Bangladesh (Al Banna *et al*., 2020) and Taiwan (Wong *et al*., 2020) showed high prevalence of anxiety and depressive symptoms as well.

In Europe, several waves of the UK Household Longitudinal Study conducted between 2018/2019 and April 2020 (i.e. after approximately 1 month of lockdown in the UK) were compared, and it was demonstrated that the prevalence of clinically significant levels of mental disorders, as measured by the 12-item General Health Questionnaire, increased from 18.9 to 27.3% (Pierce *et al*., 2020). A nationwide study from Italy using convenience sampling (*n* = 500) reported that 19.4 and 18.6% of participants experienced mild and moderate-to-severe psychological distress respectively (Moccia *et al*., 2020). In Spain, respondents (*n* = 3480) of an online survey reported high prevalence of depression (18.7%), anxiety (21.6%) and post-traumatic stress disorder (15.8%) (González-Sanguino *et al*., 2020). High prevalence of depression (23.6%) and anxiety (45.1%) were also found in respondents (*n* = 343) of an online survey in Turkey (Özdin and Özdin, 2020). The nationally representative online survey which reported a baseline comparison comes from Denmark (*n* = 2458), where the WHO-5 Well-being Index was utilised finding that the Danish population, and especially females, reported lower emotional well-being during the pandemic than in 2016 (Sønderskov *et al*., 2020). In addition, recently published data from the United States demonstrated elevated levels of mental health problems among US adults; the presence of both, anxiety or depressive symptoms was about three times higher in June 2020 than in the second quarter of 2019, and substance use and suicidal ideation was elevated as well (Czeisler *et al*., 2020).

While the evidence suggests that COVID-19 is affecting population health negatively, no existing study has measured the prevalence of COVID-19-related mental illness on a nationally representative sample using an established psycho-diagnostic instrument, and existing studies lack a reliable baseline analysis against which it compares the prevalence of mental disorders to. We aimed to conduct a study aligned with the published mental health research priorities for the COVID-19 pandemic (Holmes *et al*., 2020), by assessing differences in the prevalence of current affective, anxiety and alcohol use disorders, and suicide risk screened using an established psycho-diagnostic instrument among a representative sample of Czech adults in 2017 and 2020.

## Method

### Setting

To assess the prevalence of current mental disorders in the Czech adult non-institutionalised population during the COVID-19 pandemic, we utilised data collected between 6th and 20th May 2020. In Czechia, the COVID-19-related nationwide state of emergency lasted from 12th March to the 17th of May, greatly impacting businesses and workers. During this period, services considered as non-essential by the Government of the Czech Republic were limited and citizens were under stay-at-home orders implemented on 16th March. Restrictions were gradually lifted, with businesses opening in waves according to their size and purpose (see the following reference for detailed introduction and easing of restrictions as well as for a list of essential services: Government of the Czech Republic, 2020). Since the psycho-diagnostic instrument used in this study assess whether the examined symptoms occurred in the period of last 2 weeks or more (see ‘Measurement’ for details), the obtained data reflect the period of the peak of COVID-19-related national emergency and the most severe associated restrictions within Czechia. This was the period immediately following the first peak of COVID-19 in CZ when stay-at-home orders were not in place anymore.

### Data and participants

Since face-to-face data collection was not feasible during the state of emergency, we utilised a combination of computer-assisted telephone interviewing (CATI) and computer-assisted web interviewing (CAWI). Individuals aged 18 years and older were eligible to participate. Participants were sampled via randomly emailing (CAWI) or telephoning (CATI) to individuals registered in the online database of a data collection agency, while respecting the distribution of the Czech non-institutionalised adult population. While we obtained data from 3021 respondents, 907 of them were interviewed using CATI (response rate = 43%) and 2114 completed CAWI (response rate = 93%); each of these subsamples were representative for the Czech non-institutionalised adult population in terms of gender, age, education, size and region of residence. As the representativeness was established according to the last census that was conducted in 2011, we asked the data-collecting agency to adjust the sample to be in-line with the more recent distribution of population as per the latest Demographic Yearbook of the Czech Republic (CZSO, 2019). Thus, post-stratification weights were applied to the sample. All respondents provided oral informed consent and, at the end of the interview, they were informed about the emergency hotline providing psychological aid to Czech residents during the COVID-19 pandemic. This study was approved by the Ethics Committee of the National Institute of Mental Health, Czech Republic (registration number 127/20).

For our baseline analysis, we used data from the 2017 Czech Mental Health Study (CZEMS), which is described in detail elsewhere (Winkler *et al*., 2018*a*; Formánek *et al*., 2019). Briefly, eligibility criteria were the same as in 2020 survey, but the data were collected using the PAPI method and two-staged sampling, with a random sample of participants being selected from a random group of voting districts. The response rate was 75% and the final sample of 3306 respondents was representative of the Czech general population in terms of age, gender, education and region of residence. The description of the 2017 sample is provided in online Supplementary Table 1.

### Measurement

In both the 2017 and 2020 surveys, we assessed the presence of mental disorders via the fifth version of the Mini International Neuropsychiatric Interview (M.I.N.I.), a psycho-diagnostic instrument which has demonstrated a high concordance with clinician-assessed diagnosis of mental disorders (Sheehan *et al*., 1997, 1998). We focused on the prevalence of current (as opposed to life-time) mental disorders; i.e. the presence of examined symptoms within the past 2 weeks for major depressive episode; the past month for panic, posttraumatic stress disorder, social phobia and suicide risk (low, medium or high risk); the past 6 months for generalised anxiety disorder (GAD); and the past 12 months for alcohol use disorders. For agoraphobia, no specific periods are specified in the M.I.N.I.

Since the diagnosis of alcohol use disorders require social dis-functioning (such as not going to work because of alcohol use), the chance of which is limited due to restrictions imposed during the lockdown, we measured also, usual consumption of alcohol (expressed in number of glasses per drinking session and examined separately for beer, wine and spirits) and the frequency of occurrence of binge drinking (at least five glasses of beer, wine or spirits per drinking session) in the last 12 months.

In addition, we assessed the following: consumption of prescription drugs (pain killers, sleeping pills, tranquilisers and stimulants) expressed as the number of drug categories consumed on daily basis; professional (i.e. psychiatrist/psychologist/general practitioner) mental health help-seeking in the last 12 months; COVID-19 health and economic-related worries (direct and indirect, see online Supplementary Appendix for details), expressed as the number of items with strong worries (min 0, max 2), and the presence of COVID-19 symptoms. All items were self-reported. The exact wording of COVID-19-related questions and the distribution of responses on these questions is provided in online Supplementary Table 2.

### Statistical analysis

First, we calculated descriptive statistics for the sample, expressed as counts and percentages (%) for non-continuous variables, and as means with standard deviations (SD) for continuous variables. Second, we calculated the prevalence of mental disorders for 2017 and 2020. We expressed prevalence as weighted means with 95% confidence intervals (95% CIs) estimated via bootstrap method with 10 000 replicates. To address the slightly higher proportion (approximately 2%) of women in the 2017 sample, we employed post-stratification weights for the 2017 dataset.

Finally, we performed logistic regression to assess whether COVID-19-related worries and the self-reported presence of COVID-19 symptoms were associated with the presence of (1) any mental disorder, (2) major depressive episode, (3) suicidality, (4) anxiety disorders and (5) alcohol use disorders in our 2020 sample. We controlled for age, gender, level of education, work status, marital status, size of residence and prescription drugs use in all models. We report results as odds ratios (ORs) with 95% CI, considering associations with *p* < 0.05 as statistically significant. We report the crude models adjusted for age and gender in online Supplementary Table 3. All analyses were performed using R statistical programming language (version 3.6.0).

## Results

Detailed characteristics of both November 2017 and May 2020 samples are provided in Table 1. In 2020, approximately 10% of the sample reported using at least one type of prescription drugs on daily basis, while 1% reported using three to four different types. A considerable number of individuals (6.5%) expressed strong worries on both questions regarding the health consequences of COVID-19. Additionally, 8.5% of participants reported strong worries on both questions focused on the economic consequences of COVID-19. Approximately 2% of individuals reported having been tested (either positively or negatively) for COVID-19; and about 10% of the sample had visited a health professional regarding their mental health within the last 12 months.

**Table 1.** Description of the 2017 and 2020 sample (unweighted)

	2017	2020
Gender, *n* (%)		
Males	1532 (46.34)	1440 (47.67)
Females	1774 (53.66)	1581 (52.33)
Age, mean (sd)	48.82 (17.19)	46.84 (16.02)
Education, *n* (%)		
Elementary	278 (8.41)	180 (5.96)
Other than elementary	3028 (91.59)	2841 (94.04)
Marital status, *n* (%)		
Other than married/living with a partner	1314 (39.75)	1243 (41.15)
Married/living with a partner	1992 (60.25)	1778 (58.85)
Employment status, *n* (%)		
Employed, student, retired, receiving benefits	3194 (96.61)	2917 (96.56)
Unemployed	112 (3.39)	104 (3.44)
Size of residence, *n* (%)		
0–4999	1264 (38.23)	1064 (35.22)
5000–19 999	609 (18.42)	573 (18.97)
20 000–99 999	725 (21.93)	671 (22.21)
10 000 and more	708 (21.42)	713 (23.6)
Daily use of prescription drugs – number of drugs categories, *n* (%)		
0	3133 (94.77)	2703 (89.47)
1	132 (3.99)	211 (6.98)
2	28 (0.85)	75 (2.48)
3	10 (0.3)	25 (0.83)
4	3 (0.09)	7 (0.23)
COVID-19 health-related worries – number of items with strong worries, *n* (%)		
0	NA^a^	2476 (81.96)
1	NA	350 (11.59)
2	NA	195 (6.45)
COVID-19 economic worries – number of items with strong worries, *n* (%)		
0	NA	2516 (83.28)
1	NA	251 (8.31)
2	NA	254 (8.41)
Presence of COVID-19, *n* (%)		
Not tested	NA	2961 (98.01)
Tested negative or positive	NA	60 (1.99)
Received treatment in the last 12 months		
No	3122 (94.43)	2725 (90.2)
Yes	184 (5.57)	296 (9.8)

a Not applicable.

**Fig. 1.**
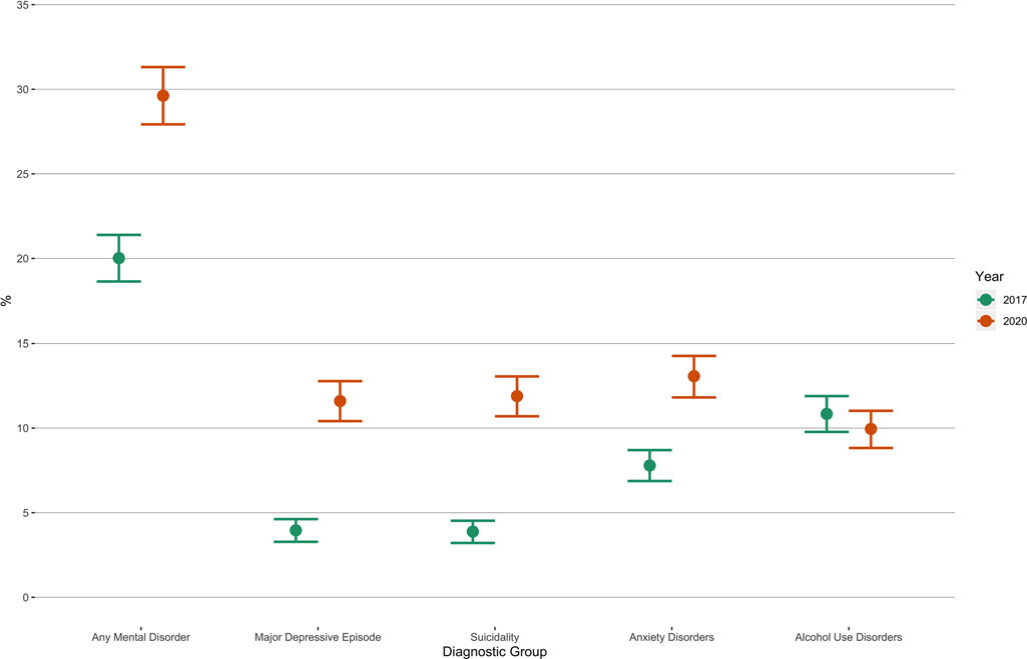
Prevalence of mental disorders among non-institutionalized adults in the Czech Republic: November 2017 and May 2020.

A detailed comparison of prevalence in 2017 and 2020 is presented in Table 2 and is graphically displayed in Fig. 1. The proportion of those experiencing symptoms of at least one current mental disorder increased during the COVID-19 pandemic by more than 10% (20.02, 95% CI = 18.64; 21.39 *v.* 29.63, 95% CI = 27.9; 31.37) when compared to the baseline in November 2017. While the prevalence of current affective disorders increased by almost 12.5% (6.57, 95% CI = 5.71; 7.4 *v.* 18.58, 95% CI = 17.09; 20.05), the prevalence of current anxiety disorders increased by approximately 6% (7.79, 95% CI = 6.87; 8.7 *v.* 12.84, 95% CI = 11.6; 14.05). The prevalence of alcohol use disorders in 2020 was approximately the same as in 2017 (10.84, 95% CI = 9.78; 11.89 *v.* 9.88, 95% CI = 8.74; 10.98); however, there was a significant increase in consumption of alcohol as measured by both, the number of glasses per drinking session for all examined beverages (beer 1.62 *v.* 1.8, wine 1.41 *v.* 1.62 and spirits 1.24 *v.* 1.32) as well as the number of individuals who binge drank at least once per week (4.07 *v.* 6.39%).

**Table 2.** Prevalence of mental disorders per study years

	2017	2020
Any mental disorder	20.02 (18.64; 21.39)	29.63 (27.9; 31.37)
Affective disorders	6.57 (5.71; 7.4)	18.58 (17.09; 20.05)
Anxiety disorders	7.79 (6.87; 8.7)	12.84 (11.6; 14.05)
Alcohol use disorders	10.84 (9.78; 11.89)	9.88 (8.74; 10.98)
Affective disorders		
Major depressive episode	3.96 (3.28; 4.62)	11.77 (10.56; 12.99)
Suicidality	3.88 (3.21; 4.52)	11.88 (10.64; 13.07)
Anxiety disorders		
Panic disorder	0.21 (0.04; 0.36)	0.88 (0.53; 1.18)
Generalised anxiety disorder	3.14 (2.52; 3.72)	5.17 (4.31; 5.95)
Agoraphobia	5.16 (4.4; 5.91)	7.99 (6.99; 9)
Social phobia	1.67 (1.22; 2.09)	2.53 (1.94; 3.07)
Posttraumatic stress disorder	0.96 (0.61; 1.28)	1.7 (1.23; 2.15)
Alcohol use disorder		
Alcohol abuse	9.42 (8.39; 10.41)	7.85 (6.85; 8.79)
Alcohol dependence	6.61 (5.72; 7.48)	4.25 (3.49; 5)

The results are expressed as weighted proportions (%) with weighted 95% CIs.

The main results of the logistic regression are provided in Table 3, and the results containing all associations in online Supplementary Table 4. Both strong worries from health and economic consequences of COVID-19 were associated with an increased risk for fulfilling the criteria of at least one mental disorder (1.63, 95% CI = 1.4; 1.89 and 1.42, 95% CI = 1.23; 1.63 respectively), major depressive episode (1.66, 95% CI = 1.38; 1.99 and 1.44, 95% CI = 1.21; 1.71), risk of suicide (1.43, 95% CI = 1.19; 1.72 and 1.37, 95% CI = 1.15; 1.62) and anxiety disorders (1.7, 95% CI = 1.42; 2.02 and 1.43, 95% CI = 1.2; 1.69). However, we found no statistically significant association between alcohol use disorders and health or economic COVID-19 worries. Having been tested (either negatively or positively) for COVID-19 was associated with elevated risk of at least one mental disorder (2.13, 95% CI = 1.21; 3.73), risk of suicide (2.36, 95% CI = 1.23; 4.32) and anxiety disorders (2.11, 95% CI = 1.08; 3.95), but not for major depressive episode or alcohol use disorders.

**Table 3.** Logit regression models: an association of COVID-19-related covariates and the presence of mental disorders

	Any mental disorder	Major depressive episode	Suicidality	Anxiety disorders	Alcohol use disorders
COVID-19 health-related worries	1.63 (1.4; 1.89)***	1.66 (1.38; 1.99)***	1.43 (1.19; 1.72)***	1.7 (1.42; 2.02)***	1.12 (0.88; 1.41)
COVID-19 economic worries	1.42 (1.23; 1.63)***	1.44 (1.21; 1.71)***	1.37 (1.15; 1.62)***	1.43 (1.2; 1.69)***	1.13 (0.91; 1.38)
Presence of COVID-19					
Not tested	Ref.	Ref.	Ref.	Ref.	Ref.
Tested negative or positive	2.13 (1.21; 3.73)*	1.75 (0.87; 3.34)	2.36 (1.23; 4.32)*	2.11 (1.08; 3.95)*	0.96 (0.39; 2.07)

Models adjusted for age, gender, level of education, marital status, employment status, size of residence and use of prescription drugs. The results are expressed as ORs with 95% CIs.

*p < 0.05; **p < 0.01; ***p < 0.001.

## Discussion

Our results confirm that the concerns expressed by experts and previous studies expressing mental health-related consequences of the COVID-19 pandemic pose a major threat to population health are real and alarming in the context of Czechia. We found an approximate 10% increase in the proportion of Czech adults fulfilling the criteria of at least one current mental disorder during the COVID-19 pandemic in May 2020 as compared to the baseline in November 2017.

The prevalence of affective disorders and anxiety disorders both increased, by 12.5 and 7%, respectively. While the prevalence of alcohol use disorders remained similar, the consumption of alcohol measured by both, number of glasses of beer, alcohol and spirits and binge drinking, was higher during the COVID-19 pandemics than prior. Strong worries related to health and economic consequences of COVID-19 were associated with considerably higher odds for at least one mental disorder, major depressive episode, suicide risk or anxiety disorders. In addition, having been tested for COVID-19, irrespective of test result (negatively or positively), was associated with a higher chance of scoring positively for at least one mental disorder and suicidality.

The sustained prevalence of alcohol use disorders at baseline and during the pandemic might partially be explained by limited possibilities of social dis-functioning during the lockdown, which is a diagnostic criterion of alcohol use disorders. For other mental disorders, the lockdown and other restrictive measures resulted in increased chances of positive scoring, because they had direct impact on population functioning and might negatively influence interest in hobbies or social activities, feelings of detachedness or isolation, feelings of tiredness, sleeping habits, or appetite, which are all symptoms used by M.I.N.I. to identify a presence of current mental disorder.

The sharp increase in the prevalence of current mental disorders supports the notion that population mental health is highly receptive to socio-economic factors. Similar phenomena have been researched in the context of the negative influence of the last financial crisis on completed suicides across Europe (Fountoulakis *et al*., 2014), and seem to be supported by emerging evidence related to the COVID-19 pandemics as well (Mamun and Ullah, 2020). The current study demonstrates significantly higher odds of current mental disorders among individuals who expressed health or economic worries associated with the COVID-19 pandemic. This supports a model of mental health problems existing along a continuum (Patel *et al*., 2018), where a considerable amount of the population experiences mild symptoms, leaving the population exceptionally vulnerable during stressful situations.

The increase in the prevalence of mental disorders from 2017 to 2020 in the Czech context should be interpreted taking into consideration the reform of mental health care and national efforts towards the deinstitutionalisation of mental health care (Winkler *et al*., 2017). As this study includes only community-dwelling participants, it might be argued that some confounding might occur because of an increased number of individuals with mental illness being based in the community. However this is largely unlikely as the target population for deinstitutionalisation are patients with psychosis (Winkler *et al*., 2018*b*), which were not included in the current study, and at most, patients with comorbidities in mental health diagnosis could be represented and contribute to the increased prevalence of people with mental illness in 2020 as compared to the 2017 baseline sample.

This study has several limitations. First, since this is not a cohort study, we could not assess whether the COVID-19 pandemic led to the development of mental disorders in individuals with no history of mental disorders. In addition, the baseline data collection was conducted in November 2017, which is about two and half year before the COVID-19 data collection, and this might increase a chance of confounding. Second, due to the extraordinary epidemiological situation caused by the COVID-19 pandemic, we were not able to collect data by means of a face-to-face interviewing, instead relying on CAWI and CATI methods. The use of these methods may have introduced selection bias as some demographics (i.e. individuals without phone and/or internet access) could not participate. Third, while assessing a presence of GAD, the psycho-diagnostic tool M.I.N.I. asks the following question: ‘Is the patient's anxiety restricted exclusively to, or better explained by, any disorder prior to this point?’ This question is intended for clinicians, and it makes sense when all of the modules of MINI are used, which was not the case in 2020. Hence, we relaxed this criterion, and this has led to a relatively high prevalence of GAD in both of our samples. Fourth, since the data were collected after the strictest lockdown measures had loosened, the full extent of COVID-related mental health problems may not be represented. Consequently, it is likely that at the peak of the pandemic and associated lockdown, mental health symptoms were even higher. In China, this was found in a study reporting the progression of mental health symptoms at multiple points over COVID-19, showing that the highest levels of mental health problems presented during the peak of virus spread and associated lockdowns (Wang *et al*., 2020*a*, 2020*b*). However, the study benefits from a considerably large number of participants, obtained using rigorous sampling, representative of the Czech adult general population. Second, we were able to compare our results to a baseline dataset collected prior to COVID-19, which measured the prevalence of mental illness using the same instrument on a similar population. Third, data collection for this study was finalised prior to the lifting of the most severe restrictions imposed by the government in response to COVID-19. Thus, the results of the study should be interpreted with existing contextual confounders within the Czech context, but are likely not biased by a return to society with new norms and regulations surrounding distancing and functioning.

## Conclusions

This study provides data showing that projections and warnings of COVID-19-related mental health problems are backed by evidence, and are justified and warrant attention and action. Our study was conducted when the Czech government was lifting up restrictive measures and heading towards the end of state-of-emergency. This time also corresponds to improving epidemiological situation in continental Europe. It is possible that the prevalence of mental disorders in the general adult population could be higher during the culmination of the first peak, since the confusion and uncertainties were strongest then. However; with the threat of a second wave of COVID-19, services will need to be assessed, adapted and scaled to both meet the increased prevalence of individuals with mental health problems, and to adhere to new societal standards which parallel the pandemic including physical distancing measures, and lockdowns. Considering the existing treatment gap for mental disorders in the Czech context ranging from 61% for affective to 93% for alcohol use disorders in 2017 (Kagstrom *et al*., 2019), the increase in mental health problems poses additional burden on national efforts for comprehensive service provision and mental health reform initiatives.

Our findings showing doubling and tripling of the prevalence of common mental illnesses, which are in line with findings from UK (Pierce *et al*., 2020) and USA (Czeisler *et al*., 2020) also emphasise an urgent need to scale up mental health promotion and prevention globally, which includes integrating strategies to change mental health care in the wake of COVID-19 (Moreno *et al*., 2020). E-mental health has proved promising in delivering effective care to populations in diverse settings across the globe and scalable e-health interventions could be a major tool in meeting the surge of people with mental health problems (Wind *et al*., 2020). Continued mental health monitoring, early identification of at-risk individuals and ensuring accessible treatment for those with mental health problems will be vital aspects in service provision (Brooks *et al*., 2020; DePierro *et al*., 2020). On-going research assessing the prevalence, severity and progress in addressing mental health of populations will be necessary to track developments and inform priorities in mitigating the effects of COVID-19-related mental health consequences (Moreno *et al*., 2020).

## Data Availability

Data are not available publicly because of government regulations; however, data will be made available upon a reasonable request. Similarly, the R code will be made available upon a reasonable request.

## Supplementary material

For supplementary material accompanying this paper visit https://doi.org/10.1017/S2045796020000888. click here to view supplementary material
