# Supplementary material for: Increase in prevalence of current mental disorders in the context of COVID-19: analysis of repeated nationwide cross-sectional surveys
Source: Epidemiol Psychiatr Sci. 2020 Sep 29;29:e173. doi: 10.1017/S2045796020000888 (PMC7573458; doi:10.1017/S2045796020000888)
Supplement: Supplementary file 1 [file S2045796020000888sup001.docx]

Supplementary appendix

Supplement to: Winkler P, Formanek T, Mlada K, Kagstrom A, Mohrova Z, Mohr P, & Csemy L. Sharp increase in prevalence of current mental disorders in the context of COVID-19: analysis of repeated nationwide cross-sectional surveys.

**Details about data collection**

*Agency*

All data were collected in 2017 and 2020 by the MindBridge Consulting agency. This agency has experiences with similar kinds or research; for instance, it conducted nationwide study on substance use realized by the National Monitoring Centre for Drugs and Addiction. More details about the agency are available at the following link: http://www.mindbridge.cz/en/

*Construction of an online database/panel*

The online panel currently contains approximately 80,000 registered and 60,000 active members, and is actively managed. Members are recruited via a range of both online and offline methods. Recruitment methods include face-to-face personal inquiries, telephone recruitment from the agency’s call center, print ads, PPC Google AdWords (Google Ads) campaigns, social networks campaigns, recruitment via affiliate programs, and snowball methods where existing panel members recommend the panel to other people. The agency states that it never recruits from databases of its clients. It also states that it maintains a diversity of the panel via online and offline campaigns focused on recruitment of specific target groups (such as elderly) in cases for which the panel contains insufficient representation of a given target group. The Internet panel meets the standards for Internet research set by the Association for Market Research and Public Opinion SIMAR. Each panel member conducts a maximum of 2 surveys per week, and a maximum of 36 surveys per year.

*Verifications of the online panel members*

The panel is verified via both, offline and online methods. The first stage of verification is conducted through telephone, where the agency calls a new member following their registration, and verifies a key registration data and several parameters from their profile. The next level of control is the offline verification of the panel member. This is done as soon as a panel member collects enough credits to receive their first payment. Panel members are compensated via vouchers sent by mail to the address specified within the panel member profile. Vouchers can be received only upon presentation of a panel member’s identity card. Authentication is also conducted via comparison of members’ IP addresses in order to eliminate duplicate registrations. "

*CAWI sampling*

The sampling frame was based on probabilistic sampling, stratified by age, gender, education, region, and size of the place of residence using a list of email addresses. For each strata, a default ID was generated for each potential participant, then all IDs were randomly shuffled. Next, a number of IDs required for each strata was calculated. The first ID to be contacted was randomly selected, and a number was calculated for subsequent recruitments, following a fraction created by the formula: # recruitments desired/ # individuals in the strata. This number was then used to recruit possible IDs for each strata. Participants were recruited via an email invitation detailing the topic of research and a deadline for participation. Replacements for non-respondents (following the initial invite and an email follow-up reminder) were made by recruiting the subsequent ID number to the non-respondent. Once the next ID successfully replaced the non-respondent, the sequence was then carried on from the replacing ID.

*CATI sampling*

Telephone numbers were randomly generated for calls to mobile phones, and the distribution corresponding to the distribution of the Czech population with regard to age, gender, education, region, and size of the place of residence was followed. Calls to mobile phones (85%) were combined with calls to landline phones (15%) located in Czech administrative districts or towns.

**Supplementary table 1 Description of the 2017 sample**

|  |  |
| --- | --- |
| Gender, n (%) |  |
| Males | 1532 (46.34) |
| Females | 1774 (53.66) |
| Age, mean (SD) | 48.82 (17.19) |
| Education, n (%) |  |
| Basic | 278 (8.41) |
| Other than basic | 3028 (91.59) |
| Marital status, n (%) |  |
| Other than married/living with a partner | 1314 (39.75) |
| Married/living with a partner | 1992 (60.25) |
| Work status, n (%) |  |
| Not unemployed | 3194 (96.61) |
| Unemployed | 112 (3.39) |
| Size of residence, n (%) |  |
| 0-4 999 | 1264 (38.23) |
| 5 000-19 999 | 609 (18.42) |
| 20 000-99 999 | 725 (21.93) |
| 100 000 and more | 708 (21.42) |
| Daily use of prescription drugs – number of drugs categories, n (%) |  |
| 0 | 3133 (94.77) |
| 1 | 132 (3.99) |
| 2 | 28 (0.85) |
| 3 | 10 (0.3) |
| 4 | 3 (0.09) |
| Received treatment in the last 12 months |  |
| No | 3122 (94.43) |
| Yes | 184 (5.57) |

**Supplementary table 2 Distribution of responses to COVID-19-related questions**

| I am afraid that I may become infected with COVID-19, n (%) |  |
| --- | --- |
| Definitely yes | 228 (7.55) |
| Rather yes | 755 (24.99) |
| Rather no | 1430 (47.34) |
| Definitely no | 599 (19.83) |
| Not applicable | 9 (0.3) |
| I am afraid that my close ones may become infected with COVID-19, n (%) |  |
| Definitely yes | 512 (16.95) |
| Rather yes | 1206 (39.92) |
| Rather no | 999 (33.07) |
| Definitely no | 262 (8.67) |
| Not applicable | 42 (1.39) |
| I am afraid that I may experience serious economic problems as a result of COVID-19, n (%) |  |
| Definitely yes | 335 (11.09) |
| Rather yes | 702 (23.24) |
| Rather no | 1296 (42.9) |
| Definitely no | 671 (22.21) |
| Not applicable | 17 (0.56) |
| I am afraid that my close ones may experience serious economic problems as a result of COVID-19, n (%) |  |
| Definitely yes | 424 (14.04) |
| Rather yes | 909 (30.09) |
| Rather no | 1214 (40.19) |
| Definitely no | 414 (13.7) |
| Not applicable | 60 (1.99) |
| Did you experience symptoms that are typical for COVID-19)?, n (%) |  |
| I did not experience any such symptoms | 2697 (89.28) |
| I experienced such symptoms, but was not tested for the presence of COVID-19 | 211 (6.98) |
| I experienced such symptoms and was tested negatively for the presence of COVID-19 | 55 (1.82) |
| I experienced such symptoms and was tested positively for the presence of COVID-19 | 5 (0.17) |
| Don’t know what are the symptoms of COVID-19 | 53 (1.75) |

**Supplementary table 3 Crude logistic regression models**

|  | Any mental disorder | Major Depressive Episode | Suicidality | Anxiety disorders | Alcohol use disorders |
| --- | --- | --- | --- | --- | --- |
| COVID-19 health-related worries | 1.63 (1.41; 1.88)*** | 1.68 (1.41; 2.01)*** | 1.44 (1.20; 1.73)*** | 1.69 (1.43; 2.00)*** | 1.12 (0.88; 1.40) |
| COVID-19 economic worries | 1.48 (1.29; 1.69)*** | 1.51 (1.27; 1.77)*** | 1.44 (1.22; 1.70)*** | 1.49 (1.26; 1.75)*** | 1.13 (0.92; 1.38) |
| Presence of COVID-19 |  |  |  |  |  |
| Not tested | Ref. | Ref. | Ref. | Ref. | Ref. |
| Tested negative or positive | 2.25 (1.31; 3.88)* | 1.96 (1.00; 3.61)* | 2.49 (1.33; 4.44)*** | 2.28 (1.20; 4.13)* | 1.06 (0.43; 2.25) |

Models adjusted only for age and gender.

* p< 0.05; ** p< 0.01; *** p<0.001

**Supplementary table 4 Fully adjusted regression models with all associations**

* p< 0.05; ** p< 0.01; *** p<0.001

|  | Any mental disorder | Major Depressive Episode | Suicidality | Anxiety disorders | Alcohol use disorders |
| --- | --- | --- | --- | --- | --- |
|  |  |  |  |  |  |
| Age | 0.97 (0.97; 0.98)*** | 0.97 (0.96; 0.98)*** | 0.98 (0.97; 0.99)*** | 0.98 (0.97; 0.99)*** | 0.96 (0.95; 0.97)*** |
| Gender |  |  |  |  |  |
| Males | Ref. | Ref. | Ref. | Ref. | Ref. |
| Females | 0.99 (0.83; 1.17) | 1.68 (1.31; 2.15)*** | 1.22 (0.97; 1.55) | 2.23 (1.76; 2.85)*** | 0.35 (0.27; 0.45)*** |
| Education |  |  |  |  |  |
| Basic | Ref. | Ref. | Ref. | Ref. | Ref. |
| Other than basic | 1.22 (0.93; 1.61) | 0.89 (0.64; 1.26) | 1.16 (0.82; 1.67) | 1.36 (0.95; 1.97) | 1.01 (0.69; 1.52) |
| Marital status |  |  |  |  |  |
| Other than married/living with a partner | Ref. | Ref. | Ref. | Ref. | Ref. |
| Married/living with a partner | 0.84 (0.71; 1.00) | 0.84 (0.66; 1.08) | 0.71 (0.56; 0.90)*** | 0.75 (0.59; 0.94)* | 1.03 (0.79; 1.34) |
| Work status |  |  |  |  |  |
| Not unemployed | Ref. | Ref. | Ref. | Ref. | Ref. |
| Unemployed | 2.83 (1.82; 4.44)*** | 2.17 (1.30; 3.53)*** | 2.48 (1.52; 3.95)*** | 2.44 (1.49; 3.94)*** | 1.32 (0.68; 2.39) |
| Size of residence | 1.05 (0.98; 1.12) | 0.99 (0.89; 1.09) | 1.00 (0.91; 1.1) | 1.03 (0.93; 1.13) | 1.19 (1.07; 1.32)*** |
| Prescription drugs use | 2.34 (1.98; 2.79)*** | 2.29 (1.93; 2.72)*** | 1.97 (1.67; 2.32)*** | 2.26 (1.91; 2.67)*** | 1.20 (0.94; 1.50) |
| COVID-19 health-related worries | 1.63 (1.40; 1.89)*** | 1.66 (1.38; 1.99)*** | 1.43 (1.19; 1.72)*** | 1.70 (1.42; 2.02)*** | 1.12 (0.91; 1.38) |
| COVID-19 economic worries | 1.42 (1.23; 1.63)*** | 1.44 (1.21; 1.71)*** | 1.37 (1.15; 1.62)*** | 1.43 (1.20; 1.69)*** | 1.13 (0.91; 1.38) |
| Presence of COVID-19 |  |  |  |  |  |
| Not tested | Ref. | Ref. | Ref. | Ref. | Ref. |
| Tested negative or positive | 2.13(1.21; 3.73)* | 1.75 (0.87; 3.34) | 2.36 (1.23; 4.32)* | 2.11 (1.08; 3.95)* | 0.96 (0.39; 2.07) |

**Supplementary table 5 Sensitivity analyses: logistic regression with binary coded COVID-19 worries**

|  | Any mental disorder | Major Depressive Episode | Suicidality | Anxiety disorders | Alcohol use disorders |
| --- | --- | --- | --- | --- | --- |
| COVID-19 health-related worries |  |  |  |  |  |
| No worries | Ref. | Ref. | Ref. | Ref. | Ref. |
| At least one worry | 1.98 (1.60; 2.45)*** | 2.09 (1.59; 2.74)*** | 1.80 (1.37; 2.37)*** | 2.15 (1.65; 2.80)*** | 1.21 (0.86; 1.67) |
| COVID-19 economic worries |  |  |  |  |  |
| No worries | Ref. | Ref. | Ref. | Ref. | Ref. |
| At least one worry | 1.97 (1.58; 2.45)*** | 2.04 (1.54; 2.68)*** | 1.84 (1.39; 2.42)*** | 1.91 (1.46; 2.50)*** | 1.28 (0.91; 1.77) |
| Presence of COVID-19 |  |  |  |  |  |
| Not tested | Ref. | Ref. | Ref. | Ref. | Ref. |
| Tested negative or positive | 2.11 (1.20; 3.70)* | 1.72 (0.85; 3.29) | 2.32 (1.20; 4.26)* | 2.07 (1.06; 3.87)* | 0.96 (0.39; 2.05) |

* p< 0.05; ** p< 0.01; *** p<0.001

Models adjusted for age, gender, level of education, marital status, work status, size of residence and use of prescription drugs
